# Surviving in the trails: teacher’s lived experiences in remote areas

**DOI:** 10.3389/fsoc.2025.1456269

**Published:** 2025-03-05

**Authors:** Mark Neil A. Galut

**Affiliations:** Apayao State College, Apayao, Philippines

**Keywords:** remote area, teachers’ lived experience, challenges, culture-based teaching, teacher resilience

## Abstract

This study examines the experiences of teachers who are assigned to teach in remote areas. This qualitative hermeneutic phenomenological research study investigates the teachers’ journeys in surviving the trails of teaching in remote areas. Using hermeneutic phenomenological research methods, such as in-depth interviews and focus group discussions, this study found that assigning teachers to remote areas or distant locations is difficult not only in Kabugao District but also across the country. The findings revealed that surviving in the trails of the lived experiences of teachers in a remote areas had diverse experiences: Accessibility at the end of the trail, teachers’ love and passion, experience is the best teacher, eagerness behind challenges, culture-based teaching, teaching is fulfilling a life-changing experience, quality education is possible through support and connection, twenty-first-century teachers and twenty-first-century IP learners in remote area. Going to their respective stations needs to hike for almost a day or ride on a boat for almost 5 h. Teachers sacrifice their own money for the welfare of their learners. They provide a valuable contribution to our understanding of the challenges and resilience of teachers working in remote areas. Teachers of this study suggest increasing the support for the next teachers who are assigned to teach in remote areas and include the provision of adequate resources and infrastructure, as well as the development of support networks to improve their working conditions.

## 1 Rationale

Teachers play a vital role in shaping the future of our society. Accordingly, they are the assets of the community as they serve as living catalysts to uphold values, instill dignity, develop integrity, and protect the rights to education of every individual (MacDonald and Weller, 2017). It is important for teachers to be positive role models for their students. They provide them with the guidance and support they need to succeed and become productive citizens.

It is important to consider the welfare of teachers when making decisions about education policy. As stipulated in R.A.4670 or the Magna Carta for Public School Teachers (Llego, 2019), the public school teachers in the Philippines are mandated to devote actual work day. Equipado and Gilbas (2021) cited in the study of Martinez and Yap (2017) who enumerated the challenges on basic education. They mentioned that responsive education can be best attained if the Philippine Development Plan would be properly implemented for better performance of teachers. Their study finds an ally in the paper of Marquez (2017) who recommended that critical thinking in Philippine education should also be supported, including critical pedagogy.

While there is an undeniable need for improvement in the educational system, it is also necessary to reflect on the welfare of teachers when making decisions about education policy as an important factor in the teaching and learning process. After all, there were countless stories about the sacrifices of teachers who were assigned to far-flung areas wherein, according to Regalado (2020), are great manifestations of their struggles, love for work, and for some instances devoted their time and made self-sacrifices to fulfill their commitment in the delivery of learning (Equipado and Gilbas, 2021). Furthermore, there are still many unrevealed and unpublished stories to be told about how teachers survive in the trails of remote areas.

In recent years, an increasing number of newly hired teachers are being assigned to teach in remote areas. However, teaching in a remote areas can be a challenge for teachers especially if they are not familiar with the area and its resources.

In this study, we will uncover the experiences of teachers who have been assigned to teach in remote areas. We will discuss the challenges they face and the strategies they use to survive the trails. We will also learn about the support they receive from their school district and local government unit (LGU).

Teaching in a remote areas can be a challenge for teachers. The first challenge facing teachers who are assigned to teach in remote areas is simply getting to their assigned school. This can be a difficult and time-consuming process, especially if the school is located in a remote area. Once they arrive at the school, teachers must then deal with the challenges of living in a remote area. These challenges can include finding housing, getting access to resources, finding someone to talk to especially if they are not well-versed in the language, accessing cell phone and Internet signals, and dealing with the isolation of living in a remote area.

Another challenge facing teachers in remote areas is the lack of resources available to them. This can include a lack of textbooks, instructional materials, and technology. This lack of resources can make it difficult for teachers to effectively teach their students.

Finally, teachers in remote areas often face a lack of support from their school district and local government unit (LGU). This lack of support can make it difficult for teachers to get the help they need to be successful in their jobs.

Despite the challenges, there are some teachers who have been able to find success in teaching in remote areas. These teachers have often been able to develop creative solutions to the challenges they face. They have also been able to form supportive relationships with stakeholders, private organizations, colleagues, and students.

If you are a teacher who has been assigned to teach in a remote area, there are some things you can do to find success. First, it is important to be prepared for the challenges you will face. This means being familiar with the resources available to you and the challenges you may face. It is also important to develop a support network of stakeholders, private organizations, colleagues, and students. Finally, it is important to be creative in your approach to teaching in a remote area.

### 1.1 Theoretical framework

This study is viewed from the lens of Honebein, Mark Bevir, and Albert Bandura of constructivism, interpretivism, and social learning theory. A constructivist philosophical paradigm is an approach that claims that humans construct their understanding and knowledge of the universe by experiencing things and reflecting on them. People build or construct much of what they learn via experience, according to the analogy or foundation. As a result, the constructivist believes that creating meaning is the only type of learning available. This nullifies the conventional idea of learning in a “chew, pour, and forget” manner, i.e., learning for the sake of an examination with little or no desire to use what has been learned in real-life situations (Adom et al., 2016).

Furthermore, the constructivist teacher might use instructional methods that are learner-centered rather than instructor-centered to drive the teaching and learning processes. Reciprocal questioning, jigsaw classroom, and structured controversies are some of the teaching techniques suggested by Woolfolf (2010).

On the other hand, interpretivism is a philosophy that opposes the objectivist belief that meaning exists outside of the mind in the universe. It is linked to idealism and is used to gather together many perspectives, such as social constructivism, phenomenology, and hermeneutics. Furthermore, interpretivism research focuses on the meaning and can take several techniques to reflect different aspects of the situation (iNtgrty, 2016).

Education is a process that lasts for life. This begins the day of their birth and finishes the day of their deaths. Present in all cultures, it comes in many forms, ranging from “hard knocks education” or learning by practice to formal institutional learning, from rural to urban environments, and from youth to the elderly. John Dewey’s philosophy, “Education is not life-preparedness, but Education is life itself (Deporos, 2015). As a result, effective teaching necessitates teachers possessing the skills and knowledge necessary to engage students in active and meaningful learning experiences and manage well-functioning classrooms in which students can work productively (Larkin, 2019).

## 2 Review of literature

This chapter presents a review of the Magna Carta for hiring public school teachers, conceptual and research literature, and studies, both foreign and local, related to the challenges encountered and coping mechanisms of newly hired teachers assigned to far-flung schools.

### 2.1 Magna Carta for hiring public school teachers

Education is one of the primary mainstays of advancement and improvement of any country. Hence, the State perceives the significant job and commitment of teachers in sustaining future pioneers in open help. Teachers take upon themselves the obligation of dealing with their understudies in school past the normal instructing hours. Besides, educators especially those positioned in mountain areas where there are no open help vehicles, and the best way to arrive at these zones are to walk, and hike. In acknowledgment of these brave deeds, the State will offer respect to government-funded teachers by permitting the hanging of the Philippine banner on their coffins. The State perceives the responsibility, devotion, and penances of government-funded teachers as proven by R.A No. 4670 or the “Magna Carta for State-funded Teachers.” In any case, the current arrangement needs corrections to meet the present needs of the nation’s government-funded public school teachers.

According to R.A No. 4670 s. 1, the state is hereby declared to be the policy of this Act to promote and improve the social and economic status of public school teachers, their living and working conditions, their terms of employment, and career prospects in order that they may compare favorably with existing opportunities in other walks of life, attract and retain in the teaching profession more people with proper qualifications, it being recognized that advance in education depends on the qualifications and ability of the teaching staff and that education is an essential factor in the economic growth of the nation as a productive investment of vital importance. As stated in The Magna Carta for Public School Teachers (2016), Sec. 3, recruitment policy with respect to the selection and appointment of teachers shall be clearly defined by the Department of Education; provided, however, that effective upon the approval of this Act, the following shall constitute the minimum educational qualifications for teacher-applicants; kindergarten and elementary grades, Bachelor’s degree in Elementary Education (B.S.E.ED); for teachers of the secondary schools, Bachelor’s degree in Education or its equivalent with a major and a minor, or a Bachelor’s degree in Arts and Science with at least 18 professional units in Education, and for teachers of secondary vocational and 2 years technical courses, Bachelor’s degree in the field of specialization with at least 18 professional units in education.

Provided, further, that in the absence of applicants who possess the minimum educational qualifications as hereinabove provided, the school superintendent may appoint, under temporary status, applicants who do not meet the minimum qualifications; Provided, further, that should teacher-applicants, whether they possess the minimum educational qualifications or not, be required to take competitive examinations, preference in making appointments shall be in the order of their respective ranks in said competitive examinations: moreover provided, finally that the result of the examinations shall be made public and every applicant shall be furnished with his score and rank in said examinations.

According to Quejada and Orale (2018) and Lortie (1975), the portion of the previous thought processes in educating as a calling incorporates such requirements for relational relations, serving others, adhering to well-known school propensities and material advantages of the activity; and the time similarity with family requests. In the Philippines, student-teachers thought processes in picking the instruction profession are practically comparable. A study by Hao and de Guzman (2007) abridges these thought processes as optimistic, transitory, formative, work security and strength, matchless quality, freeing, charitable, and never-ending. In the school year 2016–2017, one in every five understudies in school took up educator training (Commission on Higher Education, 2017). This is the second most bought-in program in the nation. Nonetheless, not all who are selected instruction long for turning into an instructor. A considerable lot of them were affected by individuals around them or constrained by conditions. A portion of the individuals who were educated did not think about the calling as their first decision. Many have apprehensions about the calling from the outset.

Quejada and Orale (2018) said that it is a practice in the Philippines that neophyte teacher is assigned to less attractive places, such as far-flung or mountain schools. In some cases, the desire of new teachers to gain employment for economic reasons is most of the time the main reason why neophyte teachers accept teaching jobs in far-flung places.

Mountain schools are hard to reach and frequently perilous. Venturing out to and from the closest open street requires resilience and courage. This is most similar to the motivation behind why more youthful instructors are the ones doled out to it.

A neophyte teacher given a first expert training undertaking is as of now a tremendous test. This is additionally bothered when the educator is allotted in a remote or mountain school, a long way from all conceivable solace. Nourishment, convenience, security, and wellbeing are a portion of the underlying trepidation of new educators.

The study by Ashiedu and Scott-Ladd (2012) suggests that the various extrinsic incentive schemes offered by the Department of Education and Training may have some impact on teacher attraction and retention, the intrinsic motivators, such as enjoying working with young people, intellectual fulfillment the satisfaction of contributing to society and providing positive role models to young people also play a significant factor in attraction and retention (Young, 2017).

### 2.2 Conceptual literature foreign

#### 2.2.1 Challenges encountered

A novice teacher has to be able to multitask and cope with a myriad of dilemmas that take place in the classroom with all students (Darling-Hammond et al., 2016).

One of the struggles in the part of new teachers assigned to far-flung schools is that they are having a hard time because children in rural areas may be considered more difficult to educate and have lower educational participation.

First, the opportunity costs of attending schools are often higher because many rural households are dependent on their children for help at busy times of the agricultural year such as harvest time.

Second, parents in rural areas often have a lower level of education and may attach a lower value to schooling.

Third, even where parents place a value on schooling, they may be less able to help their children learning. Parents in rural areas are less educated themselves, so they have less ability to provide support for their children. Furthermore, homes in rural areas are often ill equipped to meet the needs of children to study and often lack facilities like electricity (OECD, 2005).

Communication is also an issue especially if you are communicating with people who have different languages. Teachers have an increasingly difficult job trying to communicate effectively to classrooms that are growing in size and may contain students who come from varied backgrounds (Ministry of Education, 2015).

Moreover, the burden to the part of the newly hired teachers dealing with the students in the far-flung area is adjusting to the misbehavior of the students. According to Kelly (2015a), most of the time, these problem behaviors will not cause major disruptions. The earlier one can stop a child from misbehaving, the more likely it is that a major disruption will not occur.

The Bill of Rights (Act No. 108 of 1996, Section 24) states that every person has the right to an environment that is not detrimental to his health or wellbeing. Teaching and learning cannot take place in an unsafe environment. The most remote schools should always receive priority. Even providing solar electricity would already be a great improvement for many teachers. This is to help teachers lessen their problems in far-flung areas.

On the other hand, being a teacher with students who regularly misbehave can be a troubling aspect of the academic world. It can cause the teachers to lose hope with their students and ultimately become unhappy with their chosen job. Fortunately, there are ways in which a teacher can encourage his/her students to behave in and outside of the classroom, without simply sending them to the principal’s office or to detention. Moreover, it all starts in the classroom (Bailie, 2016). With this situation, teachers assigned to far-flung areas are struggling on how to make children learn.

Multigrade schooling is a worldwide phenomenon in most of the rural areas. Multigrade teaching is a result of financial constraints, non-availability of teachers, or lack of resources. Multigrade classes are commonly found in sparsely populated areas in rural settings or rural educators, and multigrade instruction is not an experiment or a new educational trend, but a necessity imposed, in part, by economic and geographic conditions. In an environment dominated by graded schools, the decision to combine grades can be quite difficult, especially if constituents feel shortchanged by the decision. Nonetheless, recent proposals for school restructuring reflect renewed interest in multigrade and small-scale organizations generally. Such work may eventually contest the norm of the graded school.

According to Jones (2015), the nature of these classes is in such a way that the teacher divides the time for a training session into the number of grades in the class, and students are involved in self-learning, peer-learning, and individual and group teaching–learning processes more than they are exposed to teacher’s teaching. In such cases, the teacher does not have enough opportunity for grade-to-grade training, whereas in single-grade (regular) classes, students are the same in a grade and similar to each other in terms of age and sex having fewer differences in terms of abilities and skills and a full-time teacher is assigned to teach them (Mortazavizadeh, 2014).

#### 2.2.2 Coping mechanisms

New teachers are in the process of coping up not only to their environment but also to the teaching process as well. Newly hired teachers assigned to far-flung areas had troubles. However, their struggles do not hinder them from being successful in their profession. They have different strategies in coping with the situation they are facing. A study by Wanjohi (2014) revealed that teachers used both action-based mechanisms like getting to know the individual pupils.

However, most of the mechanisms employed appeared to have been emotion-based mechanisms like discussing the problem with friends. We all react differently to changes in our lives. Changes are inevitable because nothing stays the same. Some changes are harder to cope with than others. What is important is that when we are struggling with how we are feeling, we need to be able to ask for help and talk to someone.

Everyone faces challenges from time to time, and we cannot feel happy all the time (Samaritans, n.d.). This indicates that asking for help can help teachers to deal positively with their problems. The Samaritans added that sometimes we use coping strategies we know and have used before, at other times we need to ask for help. If teachers feel lonely or isolated or are going through a difficult time, they must reach out to someone for support. This support could be their co-teachers, their family, or even their students.

In addition, people make decisions every day. Plato said that “a good decision is based on the knowledge and not on the numbers.” It is a quality of a great leader to make good decisions and to live with it. Some people are born with this skill; however, most of us are incapable of making decisions and sticking by them (Bernier, 2016). Candela et al. (2022) emphasizes the importance of being firm in our decisions. If we already decide on something, we should not let anyone destroy it, especially if that is what we think is the best for them.

On the other hand, to other teachers, being assigned to a far-flung area is against their will. Others say it is their destiny. Others even blame God. These life experiences and all the things that influence us are moving us in a certain direction that we are meant to go (Tan, 1989). Part of the real beauty of our life is that it is unpredictable. We do not know what will happen to us because life is full of surprises. We need to develop the habit of looking at whatever happens through a positive mindset instead of a negative, defeatist one (Anonymous, 2016). What we need to do is accept our fate or destiny. Arthur Rubinstein (as cited by Anonymous, 2016) said that there is no formula for success except an unconditional acceptance of life and what it brings.

#### 2.2.3 Teachers’ performance

According to Darling-Hammond et al. (2016) attrition does have variations specifically with regard to teacher preparation. The less preparation a teacher has received prior to entering the field, the more likely this will lead to a rate of two to three times as high attrition than with candidates who finished their preparation program before teaching.

Another aspect of teachers’ performance related to their availability is their ability to provide guidance and counseling to their students. This involves helping students’ whole-person development and helping students with problems concerning their academic and social life (Lai-Yeung, 2014).

Another aspect of teachers’ performance that was studied was their attendance and participation in staff meetings. Staff meetings are important for teacher professional development as they provide an avenue for teachers to share experiences and discuss issues with colleagues (Jung et al., 2015; MacDougall and Drummond, 2005).

Additionally, teachers are given an opportunity to sharpen their conversation skills, which is key to enhancing children’s learning achievement.

As new teachers experience things, they learn at the same time. They learned how reading is important because it develops the mind (Davies, 2014) and is firm in their decisions. In addition, a teacher should never stop learning because learning is growth (Kim, 2016). As they experience struggles, it is best to consider themselves lucky to experience those things. Churchill once quoted, “You never can tell whether bad luck may not, after all, turn out to be good luck.” Teachers should be thankful for these experiences. Teddy Roosevelt (as cited by Schneider, 2015) said that we should not focus on what we do not have and do not focus on what our friends do have therefore, we should be thankful all the time.

Establishing relationship is an important value that teachers should consider because everyone has a role to play so the more people involved, the more exciting and successful the teaching process can be (ISLK, 2015). It is important to have PTA meetings to involve parents in their child’s learning (Nyarwaya as cited by Kabeera, 2014) and establishing relationship with co-teachers and other people around. New teachers in far-flung areas develop also the value of having patience because it is not something that comes naturally to us (Kiam, 2014).

Newly hired teachers assigned to far-flung areas may face different struggles and hardships in life, but these experiences lead them to become more competent teachers.

### 2.3 Local: challenges encountered

It is a practice in the Philippines those neophyte teachers are assigned to less attractive places, like far-flung schools. In some cases, the desire of new teachers to gain employment for economic reasons is most of the time the main reason why neophyte teachers accept teaching jobs in far-flung places. Far-flung schools are difficult to reach and often dangerous. Traveling to and from the nearest accessible road requires stamina and courage. This is most likely the reason why younger teachers are the ones assigned to it.

One of these problems is living in a culture that is different from their own, adjustment when they move from one culture to another, being homesick, dealing with the pupils, and being underpaid and treated poorly in the far-flung areas. According to Weinstein (2015), transportation is another big problem. Kids and teachers walk several kilometers a day to school. Teachers are in demand. However, only a small number of teachers want to be assigned to far-flung areas.

It becomes more challenging if the weather is not good. They will resort to hiking as the river is too risky to pass through, and hiking is a struggle for them: the sticky mud, jumping over canals and some peaks in the mountain, trekking the different terrain, wallowing in creeks, and walking on corn fields and coconut fields with perspiration dripping from their forehead while bringing their food supplies and other needs for the entire week or a month. Despite the situation, teachers in far-flung areas have not ceased from constantly wearing cheerful faces and optimistic outlooks for the love of work and to inspire the lives of the children who are patiently waiting for every start of the week for their love and care, and that somehow change their lives through education.

Currently, a diploma is greatly needed in the marketplace. Schools are built everywhere. Even remote and mountainous areas have a school. With these demands, there is also an increase in students taking up education courses.

Teachers are the ones who are willing to sacrifice their time, money, social needs, and others just for their students. They are the ones who facilitate learning and serve as a dispenser of knowledge. Teachers are also the reasons that make people what they are today. One quote says “if there is no teacher, there is no professional.”

Teachers are the lifeblood of any education system. Schools, books, and classrooms are useless without teachers.

Their first year of teaching was a challenge. An article says that there are phases of first-year teaching. The first phase is the anticipation phase. It begins during the student teaching portion of pre-service preparation. In these phases, teachers are excited and anxious about their first teaching position. In the first week of school of newly hired teachers, excitement filled them. Survival is the second phase. This phase states that school is overwhelming for newly hired teachers in the first month of teaching. They are learning at a rapid pace but encounter many problems.

In addition, one of the most painful aspects of leaving home is that your family stays behind. They will always love you, but you are no longer a day-to-day part of their life (Ronnie, 2014).

#### 2.3.1 Coping mechanisms

When one is developing coping strategies, he/she is able to build resilience. They are able to see things from a better perspective, and they would feel much better about how they handled a certain situation. Being able to cope with things makes you a stronger person. In addition, distracting oneself and getting involved is one of the emphasized strategies for teachers as well as another person when dealing with the struggles they encounter (Factsheet, 2016).

Everyday is also an experience, and every experience is an opportunity for learning. Kim (2016) said that learning is growth; therefore, we should never stop learning. He added that the more ideas teachers culminate in their minds, the more their view of the world expands. He emphasizes that the more teachers learn, the more inspired they will be. As new teachers undergo certain changes in their life being assigned in the far-flung area, some of them think how unlucky they are. However, for others, certain changes is an opportunity. So they considered themselves lucky. Our experience of life usually has more to do with what we focus our attention on than it does with events. One bad thing might happen, but 20 good things are all around us. If we only see the bad things, then life will be bad (Davenport, 2016).

Teachers in the far-flung area should be thankful to experience such difficult situations. To be more grateful in life means that you are also allowing yourself to be happier, more contented, and more satisfied with everything that has been going on around you (Laroya, 2014). Teddy Roosevelt (as cited by Schneider, 2015) said that we should not focus on what we do not have and do not focus on what our friends do have. He continued his wisdom saying, “Focus instead on where you are, what you do well, and who you are in this very moment. Be thankful for that.”

As teachers experience many challenging situations, some are thankful and consider themselves lucky.

In addition, establishing a relationship is an important value that teachers should consider. He or she must develop a connection to his or her environment, above all, a connection to his or her students. In dealing with students, “patience is a virtue.” It is the most important quality that a teacher should have because a great teacher is very patient with their students and their parents to deal with every situation. Teachers never give up on their students and would try out new ways to help their students succeed in school (Voki, 2014).

As a teacher, it is important that you go out of your way to show your students that you like and value them (Boynton and Boynton, 2016). If they feel uncomfortable, dealing with them would be difficult. When correcting a student’s behavior, it is always more effective to give options rather than making demands. Sometimes you can get so frustrated and fed up with a difficult student that you want to hand down a severe consequence for even a minor offense (Boynton and Boynton, 2016).

Newly hired teachers assigned to far-flung areas may face different struggles and hardships in life, but these experiences lead them to become more competent teachers equipped with a unique blend of different skills needed to train the future citizens of the Philippines.

#### 2.3.2 Teachers’ performance

Teachers are assigned to the study locale; far-flung schools are usually neophytes to teaching, young but dedicated, committed, and passionate. They look at their current assignment as temporary and will eventually be re-assigned to a much better school. The lived experiences of teacher-participants are consistent with other teachers’ experiences in GIDA areas of the Philippines.

It is characterized by the poverty of the school itself, lower student competencies, and poverty-stricken communities. The poor state of schools in terms of teaching and learning resources forces teachers to slice part of their salary to support classroom activities in their desire to deliver better education (Brillantes and Nebria, 2021). Teachers need to ride relatively less safer mode of transportation and walk kilometers to reach their working stations (Quejada and Orale, 2018).

The feeling of a far-flung teacher is fulfilling but is looking forward to better assignments closer to their homes someday. They dream of many good things for their students and the community as a whole. Larger support for far-fling schools, their students, and the communities the school serves is very necessary.

### 2.4 Challenges encountered

Teachers assigned to remote areas face significant challenges that impact their performance and the quality of education delivered. According to the 2015 Education for All Global Monitoring Report, there is persistent inequality in education, with millions of students leaving primary school without acquiring basic skills, highlighting systemic issues in learning quality (UNESCO, 2015). Teachers often encounter poor working conditions, including heavy workloads, large class sizes, multigrade classrooms, and role ambiguity, all of which contribute to professional dissatisfaction (Singh and Sarkar, 2015). These schools frequently lack adequate instructional materials and facilities, forcing teachers to use their own resources to meet classroom needs (Seameo Innotech, 2013). Additionally, low salaries, limited career advancement opportunities, and bureaucratic challenges further hinder teacher motivation (Singh and Sarkar, 2015). Accessibility is also a significant issue; many teachers endure long and unsafe commutes, often using “banka” boats, motorcycles, or even animals to traverse difficult terrains (Barcena, 2018).

The school environment plays a critical role in teacher satisfaction and performance. Moore (2014) emphasizes that a positive school climate—characterized by supportive administration, clear rules, and cooperative staff—can enhance teacher satisfaction. However, many remote schools fail to provide this supportive environment, compounded by inadequate housing and safety concerns (UNICEF, 2014). Teachers also contend with socioeconomic challenges within the communities they serve. Many of these communities are impoverished, with parents having low educational attainment. Students often travel long distances to school, affecting their attendance and academic performance (Reliefweb, 2014; Peccei and Warner, 1976). Multigrade classrooms, which are common in remote areas due to low enrollment and teacher shortages, further complicate teaching, especially when addressing the needs of slow learners and non-readers (DepEd, 2009; Caoyanan, 2014).

Despite these challenges, teachers in remote areas play a pivotal role in fostering community development. They serve not only as educators but also as role models and agents of change, promoting education, values, and societal transformation (Philippine Information Agency, Barcena, 2018). To improve the situation, it is essential to enhance support systems through increased funding for housing, instructional materials, and professional development (UNICEF, 2014). Addressing transportation and safety concerns, along with providing better salaries, can improve teacher retention in remote areas. Strengthening ties with local communities and expanding educational infrastructure to reduce geographical isolation are also critical steps toward achieving equitable education. These challenges and recommendations underscore the need for holistic reforms to improve the conditions of teachers and the quality of education in remote areas.

#### 2.4.1 Coping mechanisms

Teachers assigned to remote areas employ various coping mechanisms to navigate the challenges of their profession. A crucial strategy is fostering self-belief and maintaining faith in their abilities, as Thomas Aquinas emphasized, “To one who has faith, no explanation is necessary. To one without faith, no explanation is possible.” Faith enables teachers to persevere through difficulties, even when circumstances do not go as planned (Wanderlust Worker). Professional development is equally vital, as effective training and continuing education empower teachers to handle challenges and avoid failures (Kelly, 2015b). Engaging in workshops, conferences, and teacher preparation programs provides educators with the knowledge and experience they need to excel in their roles (Hill, 2016). Additionally, the connection between teachers and students is fundamental. As Phillips (2014) noted, this bond fosters student empowerment and success while also inspiring teachers in return (Teach.com, 2016).

Home visitation is another effective strategy, enabling teachers to build connections with students and their families. De la Rosa (2014) described home visits as opportunities to bridge cultural gaps, enhance parental involvement, and improve students’ academic achievements. By observing students in their home environments, teachers gain a deeper understanding of their backgrounds, which positively influences classroom learning (Ernst-Slavit and Mason, 2011). Teachers also emphasize the importance of reading, as it develops the mind and helps children focus on communication (Davies, 2014). Promoting reading habits among students is an essential part of their academic growth, as Allington highlighted, “The best way to become a better reader is to read more.”

Collaboration with parents through Parent–Teacher Association (PTA) meetings further supports education in remote areas. These meetings serve as platforms for discussing educational issues and encouraging parental involvement, which research shows significantly improves student outcomes (GreatSchools, 2016; Kabeera, 2014). Parents’ active participation in school affairs is crucial for their children’s holistic development (Nyarwaya, as cited by Kabeera, 2014). Teachers also benefit from positive relationships with their colleagues, which foster a supportive work environment. Strong camaraderie among teachers contributes to a cohesive school atmosphere, ultimately benefiting students (We Are Teachers Staff, 2016).

Finally, teachers draw strength from reframing challenges as opportunities for growth. Churchill’s insight, “You never can tell whether bad luck may not, after all, turn out to be good luck,” reminds educators to view difficult situations as potential blessings (Davenport, 2016). By maintaining faith, fostering relationships, and continuously improving their skills, teachers in remote areas can overcome obstacles and fulfill their mission of inspiring and educating their students.

#### 2.4.2 Teachers’ performance

Teachers assigned to remote areas face numerous challenges that impact their performance. Wandira Kaggwa et al. (2015) highlighted that the performance of teachers in rural public primary schools is deteriorating, characterized by absenteeism, inadequate lesson preparation, and rote teaching. Managing teachers in these areas is particularly difficult due to factors such as higher absenteeism rates. In Uganda, for instance, rural teachers often spend fewer hours in the classroom, prioritizing private work like gardening to supplement their incomes. This issue is compounded by the physical remoteness of schools, which often necessitates lengthy travel to collect salaries. In Lesotho, rural teachers may be absent for up to 3 days to collect their pay, leaving schools understaffed during this time. Efforts to pay teachers through bank accounts aim to mitigate this problem, though challenges like limited banking infrastructure persist (Mulkeen and Chen, 2008).

Rural postings also discourage professional advancement. Urban areas provide better access to further education and developmental activities, while rural teachers must navigate limited opportunities and sometimes corrupt regional educational administrations to secure their entitlements. As a result, many teachers prefer urban assignments over remote locations (Mulkeen and Chen, 2008). Teachers in far-flung areas, such as those in the study locale, are often young, newly hired, and committed despite the hardships. They view these assignments as temporary stepping stones toward more favorable postings. The poverty-stricken communities and the inadequate teaching and learning resources in these schools force teachers to allocate portions of their salaries to support classroom activities and improve education delivery (Quejada and Orale, 2018).

In the era of globalization and digitalization, the role of teachers has become increasingly dynamic. Teachers are currently expected to be facilitators, guides, and visionaries who help shape global citizens. This evolution adds complexity to the teaching profession, especially in remote areas where new teachers struggle to adapt to both their environment and teaching responsibilities. To cope, new teachers rely on their co-teachers, build resilience, and draw inspiration from their students (Gallego, 2022). They also benefit from practice teaching combined with effective mentoring, which helps them experiment with lessons and techniques more effectively (Kelly, 2015a). Despite the struggles, these teachers remain dedicated, demonstrating that challenges in remote assignments do not hinder their commitment to their profession. As one article noted, teachers should “never stop fighting until the fight is done” (Anonymous, 2016). This determination enables teachers to overcome the odds and succeed in their roles.

### 2.5 Synthesis

The varied studies and literature from books, journals, theses, articles, and Internet libraries both foreign and local were reviewed by the researcher and noted relevance to the present study. Similarities and differences were pointed out to bring insights to the researcher and the reader. Similarities can be gathered from the fact that literature and studies reviewed deal with topics that are also the concern of the present study. In the same manner, some of the variables such as age, sex, civil status, highest educational attainment, and length of service are also the concerns of the studies reviewed and of the present one. Some of the methodologies used by the studies conducted and of the present study were likely the same.

Differences, on the other hand, can be traced from the fact that the nature of research, sampling technique, and respondents utilized were different. Some were done through experiments, which is not a concern of the present study. Although there are similarities on some of the topics and variables used, differences can still be observed.

Furthermore, a review of literature revealed that there are a lot of challenges encountered and coping mechanisms of teachers. In fact, these literature studies have provided the researcher with sufficient sources of evidence, facts, viewpoints, and approaches that would serve as guidelines in the conduct of the study to come up with the most comprehensive results as possible.

This review of literature and studies greatly helped the researcher in formulating the framework of the present inquiry and guided the researcher in his journey toward the completion of the study.

## 3 Objectives/research Question(s)

This study examines the experiences of teachers who are assigned to teach in remote areas. This qualitative phenomenological research study investigates the teachers’ journeys in surviving the trails of teaching in such areas and answers the following research questions:

1. What are the lived experiences of teachers in a remote area?
2. What do teachers in the remote areas experience the challenges?
3. How do they cope with the challenge of their experiences in order to survive in the trail?
4. What insight can they share?

## 4 Procedure/methodology

### 4.1 Research design

This research study used a qualitative design in a phenomenological approach. Burns and Grove (2007) stated that qualitative research is mostly associated with words, language, and experiences rather than measurements, statistics, and numerical figures. Qualitative research refers to the inductive, holistic, epic, subjective, and process-oriented methods used to understand, interpret, describe, and develop a theory on a phenomenon or setting. It is a systematic, subjective approach used to describe life experiences and give them meaning.

Moreover, the hermeneutic phenomenological approach is selected in this study to collect data on the lived experiences of the teachers assigned in remote area and is a good option for studying the lived experiences of teachers in remote areas. This design is used to enhance the interpretation of participants’ interview transcripts, which can help elucidate meanings and assumptions that participants may have difficulty expressing. According to Corbetta (2003), phenomenological research is a type of approach in qualitative for which interviews provide an in-depth method that can grant access to deep knowledge and explanations and help grasp the subject’s perspective. Bryman (2008) posited that through interviews or face-to-face discussions, subjective and detailed personal stories can be told, with a focus on how the interviewee understands and explains different phenomena. The researcher aimed to draw out an in-depth study of the lived experiences of teachers assigned in remote areas, their coping mechanisms, as well as insights learned. Bryman and Bell (2007) defined quantitative research as entailing the collection of numerical data and exhibiting the view of the relationship between theory and research as deductive, a predilection for the natural science approach, and as having an objectivist conception of social reality.

### 4.2 Participants and sampling

In identifying the participants of the study for in-depth interviews, the researcher utilized purposive sampling. Purposeful sampling is a technique widely used in qualitative research for the identification and selection of information-rich cases for the most effective use of limited resources (Patton, 2002). This involves identifying and selecting individuals or groups of individuals that are especially knowledgeable about or experienced with a phenomenon of interest (Creswell and Plano Clark, 2011). In addition to knowledge and experience, Bernard (2002) noted the importance of availability and willingness to participate, and the ability to communicate experiences and opinions in an articulate, expressive, and reflective manner. The following were the inclusion criteria: the research participants are former teachers assigned in a remote areas specifically in Kabugao, Apayao; all of them must be a non-residents of the locality they are assigned; they are at least 2 to 5 years of elementary school teacher in a remote area. The researcher conducted in-depth interviews with the participants.

### 4.3 Research instruments

In gathering data for this study, the researcher utilized a semi-structured interview guide, which, according to Creswell (2014), serves as a concise list of high-level topics and questions intended for the interview. Typically, this guide is designed to be only one page for ease of reference during interviews. The process of creating such a guide helps the researcher focus and organize their line of thinking and questioning. Creswell emphasized that the guide is merely a framework, allowing flexibility for the researcher to deviate from the script when unanticipated but valuable lines of questioning emerge.

To ensure the validity and reliability of the study, careful attention was given to the conceptualization of the research, as well as the methods of data collection, analysis, interpretation, and presentation of findings. The adapted interview guide underwent validation by a panel of experts from the Holy Cross of Davao College and an external validator, achieving an average rating of 4.35. This validation process underscores the importance of minimizing researcher bias while maintaining the researcher as the primary instrument in data collection.

### 4.4 Data analysis

This study used the Colaizzi method of data analysis. This approach helped the researcher interpret qualitative research data, identify meaningful information, and organize it into themes or categories. The approach follows steps in data analysis. Qualitative research uses open-ended questions to uncover how people think and feel in certain situations (Shosha, 2012).

For the in-depth interview, the researcher applied Colaizzi’s steps to analyze the data collected in the interview. The researcher read the description of each person that participated in the study to gain a sense of the participants and then extracted statements with significance to the research question, such as descriptions. To analyze the significant statements, the researcher began to articulate and scrutinize what the statements mean and create themes from the meanings, and group similar categories together and organize them into themes and finally integrated the results into a comprehensive description of the topic and return to each participant for the verification of the results regarding the study on the experiences of teachers assigned in a remote area.

For the focus group discussion, the researcher applied Colaizzi’s steps to analyze the data collected in the interview during the focus group discussion for triangulation. As a researcher, there is a need to read a description of each person who participated in the study to gain the point of the participants and then extracted statements with significance to the research question, such as descriptions. To analyze the significant statements, the researcher began to articulate and scrutinize what the statements mean and create themes from the meanings and group similar categories together and organize them into themes. Finally, I integrated the results into a comprehensive description of the topic and returned them to each participant for the verification of the results regarding the study on the experiences of teachers assigned in a remote area.

Participants also review the findings of every interview session. According to Patton (2002), researchers can learn a great deal about the accuracy, completeness, fairness, and validity of their data analysis by having the participants described in that analysis react to what is described and concluded. Participant feedback confirms findings and assists in confirming that each summary of the interview session is accurate and ensures that the researcher is asking the right questions.

Using the fundamental techniques of data analysis described by Dewalt and Dewalt (2002) as reading, thinking, and writing; and rereading, rethinking, and rewriting, the researcher guarantees credible categorization, organization, and summarization of the large quantities of data obtained from this study. As the researcher developed insights into the data examined and obtained documentation to support conclusions, scholarly research was accomplished. The goal of the analysis is to develop a well-supported argument that adds to the understanding of a phenomenon, whether the understanding is phrased in descriptive, interpretive, or explanatory terms.

## 5 Discussion of results

### 5.1 Major themes on surviving in the trails: Teacher’s lived experiences in remote areas

#### 5.1.1 Accessibility at the end of the trail

Most of the teachers who took part in the study taught or assigned to remote areas such as far-flung schools. Most of them felt struggled and challenged in arriving at the school, knowing they will be assigned to the schools that are really on top of the mountains. This is most certainly the explanation that it is assigned to younger teachers. In addition to that, teachers felt a deeper connection and attachment to the learners, which made them feel the fulfillment of being a teacher, not just simply a teacher, but a teacher to IPs.

Participant 1 said,

*“When I was hired, I was deployed at Baliwanan Elementary School. It is one of the farthest school in Kabugao. It was very challenging to reach the school. From Poblacion, I need to ride on a boat to cross the Apayao river. After crossing it, I need to hike for five to six hours just to reach the school. Mahirap siya lalo na noong una kong pagpunta doon dahil lahat ng personal mong gamit eh kailangan dala dala mo. Mahirap kasi wala kang ibang option to reach the school kundi ang maglakad. Bitbit bitbit mo ang lahat ng iyong mga kagamitan, pati na ang mga groceries mo. Aakyat ka ng bundok, tas bababa, pagbaba mo dadaan ka ulit sa mga bahagi ng ilog kung saan ay magkakaroon ka ng varicose veins dahil mula sa init eh malalamigan ulit ang iyong mga paa. There were times nga na naiiyak ako dahil sa layo, hirap at pagod makarating ka lamang sa eskwelahan na pagtuturuan mo.”*

When I was hired, I was assigned to Baliwanan Elementary School, which is one of the farthest schools in Kabugao. Reaching the school was extremely challenging. From Poblacion, I had to ride a boat to cross the Apayao River. After crossing, I needed to hike for five to six hours just to get to the school. It was difficult, especially during my first trip there, because I had to carry all my personal belongings. It was hard since walking was the only way to reach the school. You had to carry everything—your belongings, including groceries. You would climb mountains, then go downhill, and after that, you’d pass through parts of the river where your legs would go from hot to cold, causing varicose veins. There were even times when I could not help but cry because of the distance, exhaustion, and difficulty of simply reaching the school where I was assigned to teach.

Participant 3 said,

*“My first travel reaching the school was very exciting and nerve wrecking for me because it is my first time to ride on a boat. I cannot move and talk because I was very nervous. I was just biting my fingers in order to hide my nervousness. Luckily bumaba yung isang teacher na magiging kassama ko doon sa school na pupuntahan ko kaya meron akong nakakausap habang nakasakay ng bangka. Sa humigit kumulang 4 na oras na nakaupo sa bangka ay ramdam ko ang sakit ng aking likuran sapagkat nakakangalay. Minsan eh mahihiya ka pang magsabi na naiihi ka kaya pinipigilan ko na lamang yung pag-ihi ko. Kapag may bababa sa mga barangay na madadaanan yun lamang yung pagkakataoon na ikaw ay makakaihi gamit ang payong na panakip ng iyong sarili sa likod ng mga matataas na damo upang di ka masilipan.”*

My first trip to the school was both exciting and nerve-wracking for me because it was my first time riding a boat. I could not move or talk because I was so nervous. I just bit my fingers to hide my anxiety. Luckily, one of the teachers who would be my colleague at the school got on the boat, so I had someone to talk to during the ride. After sitting on the boat for about four hours, my back started to hurt because it was tiring to stay in one position for so long. Sometimes, you’d feel too shy to say you needed to pee, so I just held it in. The only chance to relieve yourself was when the boat stopped at one of the barangays along the way. You’d use an umbrella for cover while hiding behind tall grass and large bushes to make sure no one could see you.

Participant 2 said,

*“Actually for me I enjoyed going to my assigned school because I am an adventurous person. But I have an unforgettable experience reaching the school. After two years of teaching there, the province made sure that all barangays are accessible through land vehicle. But unfortunately, this school was surrounded by water. That is why though you can ride on a jeepney or single motor from Poblacion to its nearby barangay you still need to ride on a boat for 30 min of hike for more than an hour depende kung gaano ka kabilis maglakad. It was my first time to do this kaya hindi ko masyadong kabisado and daanan. At dahil hapon na ako nakarating doon sa kapit-barangay ng eskwelahan na pinagtuturuan ko, wala nang motor boat na masasakyan kaya hinatid ako ng driver ng single motor kung saan ako dadaan papunta doon sa aking destinasyon. At dahil mag-isa ako ay binigyan na lamang ako ng instruction na susundan ko, ngunit sa kasawiang palad ay nawala ako sa gubat. Kaya ang ginawa ko ay bumaba ako ng bundok at binaybay ko ang kahabaan ng Apayao river. Lumangoy ako, kumapit ako sa malalaking bato sa gilid nito, umakyat, bumaba sa mga naglalakihang bato, at kumapit sa mga ugat ng puno. Buti na lamang at may charge ang cellphone ko noong mga panahong iyon kung hindi ay hindi ko makikita ang aking daanan sapagkat nakarating ako doon ng pasado alas siyete na ng gabi.”*

“Actually, I enjoyed going to my assigned school because I am an adventurous person. However, I had an unforgettable experience while traveling there. After 2 years of teaching at the school, the province ensured that all barangays became accessible by land vehicles. Unfortunately, this school was surrounded by water. That’s why, even though you could take a jeepney or motorcycle from Poblacion to a nearby barangay, you would still need to ride a boat and hike for more than an hour, depending on how fast you walk.

It was my first time doing this, so I wasn’t very familiar with the path. Since I arrived at the barangay near the school late in the afternoon, there were no motorboats available. The motorcycle driver dropped me off at the point where I needed to start my journey and gave me instructions to follow. Unfortunately, I got lost in the forest. I ended up descending the mountain and following the length of the Apayao River. I swam, clung to large rocks along the riverbank, climbed up and down massive boulders, and held onto tree roots for support. Thankfully, my phone still had a charge at that time; otherwise, I would not have been able to find my way. I finally arrived at my destination past seven in the evening.”

For all times, teaching has been regarded as the noblest profession. There will be no other occupation produced if teachers do not exist. In particular, teaching is regarded as the occupation of all occupations. Participant 1 observes that studying in far-flung classrooms is somewhat different from learning in low-lying areas and that he experiences a profound sense of satisfaction recognizing that he has become a part of the children’s learning experience,

Participant 4 said.

“Actually, if you are assigned in a remote school, distance is given and I cannot forget the experience that I had during those years that I was assigned there, especially so that lumaki ako sa probinsiya pero iba pa rin yung experience ko doon lalo na sa kanilang way of life and learning. My pupils came from the top of the mountain, they cross river, and walk for a kilometer just to reach the school and acquire learning. It is very challenging to teach them because you cannot give the common examples in the lessons that you can give because they cannot really connect to it unless you are going to localized and contextualized your lessons as teacher.”

Actually, if you are assigned to a remote school, the distance is a given. I cannot forget the experiences I had during the years I was assigned there, especially since, although I grew up in the province, my experience there was very different, particularly in terms of their way of life and learning. My pupils came from the tops of mountains, crossed rivers, and walked for kilometers just to reach the school and acquire an education.

Teaching them was very challenging because you could not use the common examples in lessons that you might use elsewhere; they could not really relate to them. As a teacher, you had to localize and contextualize your lessons to make them more meaningful for the students.

This theme proves that school accessibility: The Philippine government is working to make schools, especially elementary schools, open to all barangays. As a part of this program, approximately 94.5% of school-aged children are enrolled in elementary school (House of Representatives, 2017).

The challenges faced by teachers in remote Philippine schools are well-documented in various studies. For instance, a phenomenological study by Ravago and Villanueva (2024) explored the experiences of Junior High School teachers during the COVID-19 pandemic. The study found that, despite limited training in distance learning, teachers demonstrated commitment and resilience by engaging in remote teaching, innovating their instructional methods to compensate for the lack of physical interaction, and seeking professional development opportunities to enhance their effectiveness in remote education.

#### 5.1.2 IJSSHR

Similarly, a study by Semana (2021) highlighted the difficulties of teaching in a multigrade setup in rural areas, where educators often handle multiple grade levels in a single classroom. The research emphasized the necessity for teachers to adapt their teaching strategies to accommodate students with varying levels of subject background and literacy, underscoring the importance of localized and contextualized teaching approaches.

Furthermore, a study on the lived experiences of elementary teachers in remote schools revealed that challenges such as poverty, geographic isolation, low teacher salaries, and inadequate community amenities persistently overshadow the advantages of teaching in these areas. These factors directly affect the delivery of instruction, necessitating wellness programs focused on both physical and mental health to support teachers assigned in remote areas.

These studies corroborate the narratives of the participants, highlighting the resilience, adaptability, and dedication required of teachers in remote Philippine schools. They also emphasize the need for systemic support to address the unique challenges faced by educators in these contexts.

#### 5.1.3 Love and passion in teaching

The first reaction of teacher-participants as they are assigned to a remote area is exciting and rewarding because everyday that we have seen them improving in their lessons it tickles our hearts even though the place is too dangerous for them.

Participant 1 said,

“It was exciting because, new environment, new faces of people to meet with, and new experience to share with my family as soon as I got back home. Then it was a rewarding in the sense that during my first 2 weeks being a teacher there, I have seen their improvement. Actually, I have lots of learners who were not able to recognize alphabets (letter names and letter sounds) which is why I need to divide my class into multi–multigrade (a multigrade separating the non-reader to create a non-grader class) this is my own strategy to help my learners to improve.”

Schools must keep the best teachers in the classroom because our students’ futures are on the table. Alternatively, you should promote them to positions of greater accountability. We will need to recognize and help ineffective teachers (Lisciandrello, 2017).

#### 5.1.4 Learning through experience

Teacher-participants expressed that teaching in a remote areas is difficult, but you will learn a lot about how to deal with students from various cultures and norms (Kaggwa et. al., 2016), as.

Participant 4 said.

*“The most challenging part of being a teacher in a remote area is the survival in the trails. Aside from reaching the school by hiking for 4 to 5 h of under the sun, is teaching in a multi-grade class. May mga pagkakataon na matanda na na iyong estudyante mo tas hindi pa nakakabasa kahit CVC lang. Dahil doon you still need to accommodate him/her kasi nga we have education for all. That is why you need to think of other teaching strategies to address the needs of such learners. Actually I was able to devise my own A Ba Ka Da reading book para mag-umpisa sila from the basic eh. Kasi looking at the K to 12 curriculum it is spiral and we really need to begin with the basic kaya yun yung naisip kong strategy for them.”*

The most challenging part of being a teacher in a remote area is surviving the trails. Aside from hiking for 4 to 5 h under the sun just to reach the school, there is also the challenge of teaching in a multi-grade class. There are times when your students are already older but still cannot read even simple CVC words. Because of this, you still need to accommodate them since we uphold the principle of education for all. That’s why you have to think of alternative teaching strategies to address the needs of such learners.

Actually, I was able to devise my own A Ba Ka Da reading book to help them start from the basics. Looking at the K to 12 curriculum, which follows a spiral approach, it is essential to begin with the fundamentals. That’s the strategy I came up with to support their learning.

This theme proves that teachers sometimes say that teaching experience has taught them more than coursework. A combination of doing (action) and undergoing (reflection), both personally and in a group, seemed to promote meaningful learning from all forms of teaching experiences. (Schmidt, 2010). “Experience is the greatest teacher,” teachers also claim (Goodlad, 1984).

### 5.2 Major themes on coping with the challenge of their experiences

#### 5.2.1 Eagerness in overcoming challenges

Teaching in rural areas is very difficult. You will see your students struggling in everyday life to get an education. Walk a kilometer just to get to school and go to school even without food just to learn. Knowing this as a teacher is really hard and seems to tear the heart because we are not just a teacher, we are agents of change. Participant 2 said,

*“The most rewarding moments for me when I was teacher there was conducting a summer class for my learners. It was my first summer to spend in the locality. I was challenged that time because the two district supervisor offered me to conduct mathematics summer classes in the central school and offered to give me additional incentives. But with my eagerness to help my learners I did not accept the offer instead, I said to them that the next science and mathematics fair competition, I wanted to hear the name of my learner receiving an award for winning the math quiz bee. During that summer, I tried to experience my learner’s life at regular school days. Gumigising sila ng alas dos or alas tres ng umaga para maghanda. Madilim pa lamang ay bumababa na kami ng bundok papunta sa eskwelahan. Gamit ko ang flashlight ng aking cellphone para lamang makita ko ang daanan pababa. Grabe ganoon pala ang hirap na kanilang pinag-dadaanan tuwing may klase sabi ko sa aking sarili. Kaya naman after seeing my learners improved I proposed to the parents to have a quarters for our learners near the school so that they can concentrate from their studies, plus we could conduct after class tutorials. Pagdating ng math quiz bee competition, I was very happy because I was able to prove what I have said. My learner was able to get the second place in Math quiz bee in the district level.”*

The most rewarding moments for me as a teacher were during the summer class I conducted for my learners. It was my first summer spent in the locality, and I was challenged because the two district supervisors offered me an opportunity to teach mathematics summer classes at the central school with additional incentives. However, with my eagerness to help my learners, I declined the offer. Instead, I told them that in the next mathematics competition, I wanted to hear my learner’s name being called as an awardee for winning the math quiz bee.

During that summer, I tried to experience my learners’ lives during regular school days. They would wake up at 2 or 3 in the morning to prepare. Even before sunrise, we would start descending the mountain to go to school. I used my cellphone flashlight just to see the path down. I realized how difficult their journey was every class day, and I told myself, ‘This is the kind of hardship they go through.’ After seeing the improvement in my learners, I proposed to the parents to set up a dormitory near the school so the learners could focus on their studies and we could conduct after-class tutorials.

When the math quiz bee competition came, I was very happy because I was able to prove what I had promised. My learner won second place in the Math Quiz Bee at the district level.

Teachers, on the other hand, double as second parents to their students. They are the ones who correct the students when something goes wrong, just as they do for their children. Teachers have a huge impact on the lives of the students under their charge (Sudhakar, 2017).

#### 5.2.2 Integrating culture in teaching

Multicultural education is not a mission to be completed, nor is it an aim to be reached. Rather, according to the Report of Lough (1990) it is a method of teaching that seeks to involve all pupils, encourage cross-cultural learning, and teach positive social skills in a multicultural environment. Participants 2 & 3 said,

*“Actually, the best experience that we have there was, we are not the teachers who teach our learners but also learners. We learn from our learners, like their language. Since were not from there, were times that when we teach lessons, they cannot understand the English and Filipino term and even Ilocano. That is why we used to draw it on the board in order for them to understand the lesson. With that, they were able to tell the correct term and so we teachers need to remember it until such time that we learn their language. Sometimes we used to buy biscuits to share with them during recess time while sharing stories about their culture. The sad moments for us was, may mga pagkakataon rin na pumapasok ang mga bata ng walang baon. Makikita mo sila lalabas tuwing recess pero iinom lang ng tubig tapos maglalaro na. Kapag tag-ulan naman, kailangan mo silang pauwiin nang maaga dahil mabilis umapaw ang ilog. Maliban doon ay hindi sila makaka-uwi kapag nagkataon. Madulas ang daanan at isa pa kakabahan ka rin na guro sapagkat delikado dahil prone to landslide ang lugar.”*

Actually, the best experience we had there was that we were not just teachers to our learners but also learners ourselves. We learned from our students, especially their language. As we were not from the area, there were times when they could not understand the English, Filipino, or even Ilocano terms we used in lessons. Because of this, we would often draw on the board to help them understand the lesson. Through this, they would tell us the correct term in their language, and as teachers, we had to remember it. Over time, we eventually learned their language.

Sometimes, we would buy biscuits to share with them during recess while listening to their stories about their culture. However, there were also sad moments for us. There were times when some children would come to school without snacks. You would see them go outside during recess, drink water, and then just play. During the rainy season, we often had to send them home early because the river would quickly overflow, and they would not be able to go home otherwise. The paths would become slippery, and as teachers, we were always worried because the area was prone to landslides, making it dangerous for everyone.

It does not mean that all students need good teachers, but places such as Chicago and New York will still be able to attract people to teach and work there (Mcardle, 2019).

#### 5.2.3 Teaching as a fulfilling and life-changing experience

The majority of the teacher-participants of the study reported that teaching in remote areas is more rewarding than teaching in low-land areas. They will make distinctions, and they have worked in both low-lying and high-lying environments. Furthermore, teachers felt a stronger bond and commitment to the IP students, allowing them to experience the satisfaction of becoming a coach, not only a teacher, but a change agent. Participant 1 said,

*“Aside from being excited to teach in remote area, the thought that it is a different environment, it is also an extraordinary experience to teach there. Nakikita mo kasi yung malaking pagkakaiba ng mga mag-aaral mula sa mga nasa siyudad at sa remote area eh. It really changes the lives of your learners if you were able to inspire them and touch their lives. Yung dating mga absentee learner ko noon sila isa na sila sa mga perfect attendance. And yung mga napagraduate naming isa sila sa mga honor student sa high school ngayon. Iba yung feeling na isa ka sa mga nagpabago ng kanilang buhay.”*

Aside from the excitement of teaching in a remote area and the thought of being in a different environment, it was also an extraordinary experience to teach there. You can really see the significant difference between students in the city and those in remote areas. It’s fulfilling to know that you can change the lives of your learners if you are able to inspire them and make an impact on their lives.

For example, some of my learners who used to be frequently absent are currently among those with perfect attendance. Additionally, some of the students we helped graduate have become honor students in high school. It is an indescribable feeling to know that you were part of the reason their lives changed for the better.

In a classroom environment, the teacher is the most influential figure. They were how an individual’s life in a society could be transformed by schooling. Furthermore, even though they would be sent to far-flung countries or armed war areas, teachers consider the job in the classroom with no regrets (Campos et al., 2015).

Teaching in remote areas presents unique challenges and rewards, as highlighted by various studies focusing on the Philippine educational context. Teachers often face difficulties such as limited access to resources and the necessity to develop innovative teaching strategies to meet their students’ needs. For instance, a study on the experiences of teachers in far-flung communities in West Malungon District, Sarangani, emphasizes the importance of community involvement and stakeholder support in enhancing educational outcomes in remote schools (Zamora, 2023).

Additionally, the state of motivation and work performance among teachers working in remote areas is influenced by factors such as classroom management and the development of teaching materials tailored to their students’ needs. This underscores the necessity for intrinsic motivation and adaptability among educators in these settings (Mato and Lumapenet, 2023).

Furthermore, the integration of technology in rural Philippine schools has shown positive effects on student engagement and achievement. For example, the introduction of computers in certain rural schools led to increased graduation rates and improved National Achievement Test scores, demonstrating the potential benefits of educational technology in enhancing learning outcomes (Critical Links, 2018).

These studies collectively highlight the complexities and rewards of teaching in remote areas, emphasizing the need for resourcefulness, community engagement, and innovative strategies to overcome challenges and improve educational outcomes.

### 5.3 Major themes on the insights of teachers to share with their peers

#### 5.3.1 Achieving quality education through support and connection

Despite a scarcity of learning resources, teachers are devising new approaches to deliver the education that our students seek. Participant 3 said,

*“Due to lack of teaching resources especially in K to 12 reference materials, I need to source out in order to acquire enough books to utilize in teaching my learners. I tried to find NGO who are willing to donate used books from private schools and luckily I found one. I used my own money to get those books from other province. With those books, we were able to address the needs of our learners.”*

Teachers play an important part in enlightening and educating children so that they can become valuable members of their families. As a result, the state should put in more effort and money to keep teachers safe and well so that they can do their jobs better (Global Coalition to Protect Education from Attack, 2014).

### 5.4 Bridging gaps between twenty-first teachers and IP learners in remote areas

Even if they are teaching in the far reaches of Kabugao, the teachers of remote areas ensure that they are eligible to be named 21st century teachers (Care et al., 2018). They are developing a method to use technology in the classroom to educate them. Participant 4 said,

*“I think as a 21^st^ century teacher we must provide our learners with quality education they need. Like learners in urban schools, we must teach them how to use technology. Though there is no internet connection, we cannot just sit there without doing anything for them. I think we need to equip them with the basic skills in technology so that there will be no learners left behind.”*

Many authorities (Robinson and Aronica, 2015) who have drawn parallels between classrooms from yesteryear and today have highlighted the few differences in classroom architecture and management.

#### 5.4.1 Research implications for practices

The challenging experiences of teachers in a remote area generated eight themes: accessibility at the end of the trail, teachers’ love and passion, experience is the best teacher, eagerness behind challenges, culture-based teaching, teaching is fulfilling a life-changing experience, quality education is possible through support and connection, twenty-first-century teachers and twenty-first-century IP learners in remote areas. These themes may lead to inculcating awareness among the department division and district heads, school principals, and even teachers who will benefit from the findings of this study. This qualitative study, along with previous studies on teachers’ lived experiences in remote areas or even in rural places, backs up the premise that providing more assistance to all schools in depressed regions can improve the quality of education.

#### 5.4.2 Implications to the teacher

In a remote area, a teacher’s job is more than just teaching. They are also responsible for the safety and wellbeing of their students. This can be a daunting task, especially if the area is prone to natural disasters or other hazards.

The study interviewed teachers who worked in remote areas to better understand the challenges they faced. The study found that teachers feel a great responsibility for their student’s safety and wellbeing. They also feel a need to be prepared for anything, including natural disasters.

The study has implications for the way we train and support teachers who work in remote areas. We need to make sure they have the knowledge and skills to deal with the unique challenges they face. We also need to provide them with the resources they need to keep their students safe.

#### 5.4.3 Implications to education

There is a growing body of research that documents the lived experiences of teachers in remote areas. This research has important implications for education. First, it highlights the challenges that teachers face in terms of access to resources, support, and professional development. Second, it underscores the importance of culture-based education and the need for educators to be attuned to the unique needs of their students. Finally, the research points to the need for policies and practices that better support teachers in remote areas.

The challenges that teachers face in remote areas are numerous. They often have little access to resources, support, and professional development. This can make it difficult for them to effectively meet the needs of their students. In addition, culture-based education can be difficult to implement in remote areas. Teachers may not be familiar with the local culture and context, and they may not have the necessary resources. As a result, they may not be able to provide their students with the best possible education.

Policies and practices that better support teachers in remote areas are needed. These policies should address the challenges that teachers face and should provide them with the resources and support they need to effectively educate their students. In addition, policies and practices that support culture-based education should be developed. These policies and practices should ensure that teachers have the necessary resources and support to successfully implement culture-based education.

### 5.5 Implications to future research

The study discusses the implications of the study ‘Surviving in the Trails: Teacher’s Lived Experiences in Remote Areas’ for future research. The study found that teachers who teach in remote areas face many challenges, including the accessibility of schools, lack of resources, and difficult working conditions. The authors of the study suggest that future research should highlight how to increase the support for these teachers who are assigned to teach in remote areas and include the provision of adequate resources and infrastructure, as well as the development of support networks to improve their working conditions.

## 6 Conclusion

Teaching in remote areas presents a unique blend of challenges and rewards, as reflected in the experiences of educators assigned to such locations. The narratives of teachers highlight the resilience, adaptability, and resourcefulness required to overcome physical, cultural, and educational barriers. The logistical difficulties of traversing rugged terrains, navigating rivers, and enduring long hikes underscore the physical demands of working in remote settings. However, these challenges are met with a sense of purpose and fulfillment derived from positively impacting learners’ lives.

Teachers in remote areas face the added complexity of addressing the diverse and often foundational learning needs of their students, many of whom struggle with basic literacy and numeracy. By localizing and contextualizing teaching strategies, such as creating personalized reading materials or conducting multigrade classes, educators demonstrate their commitment to ensuring that no learner is left behind. Their efforts to adapt and innovate contribute significantly to the academic improvement and personal growth of their students, some of whom have achieved remarkable success in their educational journeys.

Furthermore, the experiences of these teachers reveal the reciprocal nature of teaching. While educators impart knowledge and skills to their students, they also learn from the learners’ unique languages, cultures, and ways of life. This mutual exchange fosters deeper connections and enriches the teaching-learning process. Despite the challenges, the dedication of teachers in remote areas serves as a testament to the transformative power of education in even the most underserved communities.

The insights gained from these experiences underscore the need for sustained support from stakeholders, such as improved infrastructure, additional resources, and professional development opportunities, to enhance the effectiveness of teaching in remote areas. Ultimately, the narratives emphasize that teaching in such settings is not merely about delivering lessons but about transforming lives and building futures.

## 7 Recommendation

The information gathered throughout the process of this study has led to the development of the following recommendations.

1. Investigate the long-term effects of teaching interventions and strategies implemented in remote areas.
2. Compare the teaching strategies, challenges, and outcomes between remote and urban schools.
3. Examine the effectiveness of existing support mechanisms for teachers in remote locations, such as professional development programs, access to resources, and community involvement.

## Data Availability

The original contributions presented in the study are included in the article/supplementary material, further inquiries can be directed to the corresponding author.
